# Chemical Cleaning and Membrane Aging in MBR for Textile Wastewater Treatment

**DOI:** 10.3390/membranes12070704

**Published:** 2022-07-12

**Authors:** Huarong Yu, Siyuan Shangguan, Chenyu Xie, Haiyang Yang, Chunhai Wei, Hongwei Rong, Fangshu Qu

**Affiliations:** 1 Key Laboratory for Water Quality and Conservation of the Pearl River Delta, Ministry of Education, Guangzhou 510006, China; huarongyu@gzhu.edu.cn (H.Y.); sgsy2112016210@163.com (S.S.); weich@gzhu.edu.cn (C.W.); hwrong@gzhu.edu.cn (H.R.); 2 School of Civil Engineering, Guangzhou University, Guangzhou 510006, China; 3 Foshan Nanhai Jinglong Investment Holding Co., Ltd., Foshan 528211, China; x591780374@163.com

**Keywords:** membrane bioreactor, textile wastewater, membrane aging, chemical cleaning

## Abstract

Membrane bioreactors have been widely used in textile wastewater treatment. Intensive chemical cleaning is indispensable in the MBR for textile wastewater treatment due to the severe membrane fouling implied. This work investigated the aging of three different membranes, polyvinylidene fluoride (PVDF), polyether sulfone (PES), and polytetrafluoroethylene (PTFE), in the MBRs for textile wastewater treatment. Pilot-scale MBRs were operated and the used membrane was characterized. Batch chemical soaking tests were conducted to elucidate the aging properties of the membranes. The results indicated that the PVDF membrane was most liable to the chemical cleaning, and the PES and PTFE membranes were rather stable. The surface hydrophobicity of the PVDF increased in the acid aging test, and the pore size and pure water flux decreased due to the elevated hydrophobic effect; alkaline oxide aging destructed the structure of the PVDF membrane, enlarged pore size, and increased pure water flux. Chemical cleaning only altered the interfacial properties (hydrophobicity and surface zeta potential) of the PES and PTFE membranes. The fluoro-substitution and the dehydrofluorination of the PVDF, chain scission of the PES molecules, and dehydrofluorination of the PTFE were observed in aging. A chemically stable and anti-aging membrane would be of great importance in the MBR for textile wastewater treatment due to the intensive chemical cleaning applied.

## 1. Introduction

The textile industry has always been a water-consuming and pollution-intensive sector [1,2,3]. Textile wastewater treatment is quite challenging due to the complex composition as well as the xenobiotic and non-biodegradable dyes, textile auxiliaries, and other chemicals [1,4] used. On the other hand, the discharge limits imposed on the textile industry are increasingly stringent, which forces plants to upgrade their wastewater treatment processes. The high price of water also encourages the reuse of textile wastewater [2,5].

Membrane technology has become an attractive alternative for textile wastewater treatment [6,7]. Membrane bioreactor (MBR) possesses the merits of small footprints, low maintenance, low sludge production, and high removal of organic and recalcitrant pollutants [1,8,9]. Nanofiltration (NF) or reverse osmose (RO) could intercept non-biodegradable chemicals or dyes and, thus, enable recycling in textile wastewater treatment [2,10]. Many research studies on MBR or NF/RO for textile wastewater treatment exist. However, most of these works focused on the feasibility of membrane processes, among which membrane fouling was the most concerning issue [1,2,5]. Due to the complex composition of textile wastewater and toxic compounds, membrane fouling has always been much more severe than for municipal wastewater treatment. However, another important issue is that membrane aging was rarely considered in the MBR for textile wastewater treatment. Membrane aging is defined as the change observed in membrane properties over long-term use [11,12]. These properties can be expressed either as the membrane’s physical and chemical characteristics (e.g., chemical composition and pore size), or as performance factors (e.g., fouling rate and membrane resistance). Therefore, the anti-aging property of the membrane product was of great importance [13]. Moreover, besides the commonly used polyvinylidene fluoride (PVDF) and polyether sulfone (PES), more stable membrane material, such as polytetrafluoroethylene (PTFE), has also been examined for textile wastewater treatment [14,15].

Chemical cleaning is indispensable in the application of the membrane process. Strong acid, caustic, and hypochlorite are usually used in chemical cleaning [12,16]. Membrane aging resulting from chemical cleaning compromises the membrane system and shortens the life span of the membrane [17]. Membrane aging usually begins with the degradation of hydrophilic additives, such as polyvinyl pyrrolidone, and then proceeds to corrode the membrane material [18,19,20]. Due to heavy fouling in MBR for textile wastewater, the membrane must be subjected to more frequent chemical cleaning to recover flux [21,22], which leads to more severe aging. However, the anti-aging characteristics of membranes fabricated with different materials under the intensive chemical cleaning of textile wastewater treatment have not been systematically investigated. In particular, the aging of the PTFE membrane has rarely been reported.

Therefore, this study aimed to investigate membrane aging in textile wastewater treatment. Commercial membranes made of different materials were examined, i.e., PVDF, PES, and PTFE. Three pilot-scale MBRs were operated for exploring aging characteristics, and batch soaking tests were also carried out to probe long-term aging properties according to the concentration × time (CT, gh/L) value calculation. Pore size, pure water flux, contact angle, surface zeta potential, functional groups, and surface morphology were analyzed for all pristine and aged membrane specimens.

## 2. Material and Methods

### 2.1. Polit-Scale MBRs for Textile Wastewater Treatment

Three pilot-scale MBRs were constructed for textile wastewater treatment. The MBRs comprised post-treatment processes after an A/O process. The pilot-scale experiment was operated for 80 days, during which different organic loadings were applied to MBRs by pumping mixed liquor from different points in the aeration tank, e.g., high loading from the inlet end of the aeration tank, medium loading from the middle of the tank, and low loading from the exit end of the tank. The quality of the influent of the MBRs, i.e., the supernatant from these three points, can be found in Table 1.

Polyvinylidene Fluoride (PVDF) membrane, polyethersulfone (PES) membrane, and polytetrafluoroethylene (PTFE) membrane were used in the three MBRs. The parameters of the three membrane modules can be found in Table 2. The PVDF membrane and PES membrane are both flat sheet membranes, and the PTFE membrane is a hollow fiber membrane.

No hydraulic backwash was applied in MBR1 and MBR2, while it was used in MBR3 every 2 h at 20 LMH for 70 s, according to the supplier’s suggestion.

In order to examine the anti-aging nature of the membrane, an online chemical cleaning was applied to the three MBRs every week using backwashing sodium hypochlorite (500 ppm) and hydrochloride acid (500 ppm) sequentially, each for 15 min at 15 LMH. Offline chemical cleaning was conducted every month, and three offline chemical cleanings, in total, were applied in each MBR. Offline chemical cleaning was operated by draining the membrane tank, then filling it with sodium hydroxide at 40,000 ppm and sodium hypochlorite at 3000 ppm (~300 total free chlorine), and soaking the membrane module for 12 h. Subsequently, the membrane tank was drained and filled with hydrochloride acid at 20,000 ppm and soaked for another 12 h.

After the pilot-scale experiment, membrane samples from five different points on the membrane modules were collected, cleaned with tap water, and subjected to characterizations. The pristine membranes were also characterized for comparison.

### 2.2. Membrane Aging Batch Test

In order to further investigate the anti-aging property of the three membranes used, they were subjected to batch aging tests in static soaking mode. The membrane specimens were soaked in the chemical cleaning solution without stirring. Two batches of the membrane were subjected to acid soaking (hydrochloric acid at 19,767 ppm) and alkaline-oxide soaking (sodium hydroxide at 39,452 ppm and sodium hypochlorite at 3000 ppm). The concentrations of the cleaning chemicals were adjusted to match the concentration × time (CT, gh/L) value in the real MBR, as shown in Table 3. Membrane specimens at the soaking time of 12.16 h, 36.48 h, 6.08 days, 12.16 days, 18.24 days, and 30.40 days were collected to simulate aging durations of 1 month, 3 months, 1 year, 2 years, 3 years, and 5 years in the chemical cleaning scenario in MBR operation. The aged membrane was collected, washed with pure water, and subjected to the following characterizations.

### 2.3. Membrane Characterization

The pure water fluxes (L/m^2^h) of the pristine and aged membranes were measured at a pressure of 0.10 MPa. Prior to the test, 0.15 MPa pressure was applied for 30 min for stabilization. The flat sheet membrane (PVDF and PES) was cut to 76 mm in diameter (with a surface area of 45.34 cm^2^) to fit the UF cell (Amicon 8400, Millipore, Bedford, MA, USA) for flux measurements [23]. The hollow fiber membrane was cut to 100 mm in length, and 7 fibers were assembled into a module (with surface area of 43.96 cm^2^) using epoxy resin [24].

Mean pore size and pore size distribution of the pristine and aged membrane were measured by using a capillary flow porometer (Porometer 3G, Ashland, VA, USA) [25].

The contact angle was measured by using a drop shape analyzer (DSA100, KRUSS, Hamburg, Germany). The average and standard deviation of 5 measurements were used to describe the membrane sample [26].

The surface zeta potential was measured using a solid surface analyzer (SurPASS 3, Anton Parr, Ashland, VA, USA). Potassium chloride measuring 1 mmol/L was used as the test solution, and all experiments were carried out at room temperature (24 ± 2 °C) [27].

Surface functional groups of the membrane were analyzed by using a Fourier-transform infrared spectrometer (FTIR, Nicolet iS50, ThermoFisher, Waltham, MA, USA) with a diamond attenuated total reflectance (ATR) accessory. Wavenumbers in the range from 4000 to 650 cm^−1^ were analyzed with a resolution of 4 cm^−1^, and 64 scans for each sample were acquired [28].

Membrane specimens were also subjected to a focused ion beam scanning electron microscope (FIB-SEM, LYRA 3 XMU, TESCAN, Warrendale, PA, USA) for surface morphology analysis and pore size observation.

## 3. Results and Discussion

### 3.1. Change of Membrane Properties in the Pilot-Scale MBR Experiment

**Figure 1:**
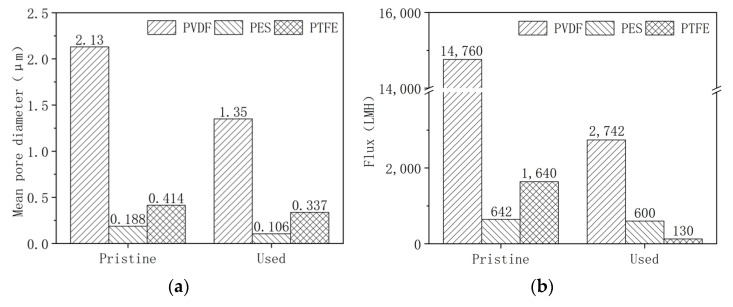

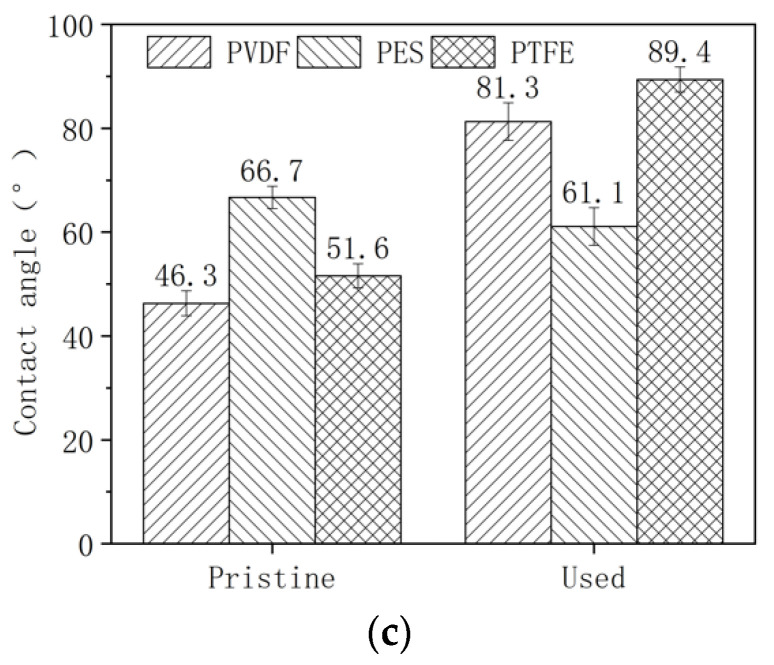
(**a**) Pore size, (**b**) pure water flux, and (**c**) contact angle of the pristine membrane and used membrane from the pilot MBRs.

Pilot-scale MBRs were operated for 80 days, during which online chemical cleaning was carried out 11 times, and offline chemical cleaning was carried out 3 times. After the experiments, the membranes in three MBRs were characterized and compared with the new membrane. As shown in Figure 1a, the pore size of all membranes decreased after being used. This may be attributed to fouling, which narrowed or blocked the pores. It should be noted that the intensive chemical cleaning adopted in the experiment may deteriorate the membrane material (the membrane aging) and, thus, enlarge the pore size, but it seems likely that membrane fouling was greater than membrane aging on varying membrane pore sizes. The pure water fluxes of the pristine membrane and used membrane also indicated membrane fouling after the MBR experiment.

The contact angles of the pristine PVDF, PES, and PTFE membrane were 46.3° ± 5.8°, 66.7° ± 6.4°, and 56.1° ± 6.2°, respectively. Usually, the PES membrane was more hydrophilic than the PVDF and PTFE membranes. The relatively lower contact angles of the PVDF and PTFE membranes may be attributed to the hydrophilic modification in the manufacture of membranes, which was always carried out for enhancing anti-fouling [29,30,31,32]. After use, the contact angle of the PVDF and PTFE membranes increased to 81.3° ± 8.2° and 89.4° ± 7.9°. Membrane aging due to chemical cleaning may undermine the hydrophilic modification of the membrane’s surface, and membrane fouling also made it much more hydrophobic. The contact angle of the PES membrane did not change significantly (from 66.7° ± 6.4° to 61.0° ± 7.6°, *p*-value = 0.14), which implied that the PES membrane may be fabricated without much hydrophilic modification. The final contact angles of the three membranes followed a common trend, e.g., PTFE > PVDF > PES, which also implied that the hydrophilic modification of the PVDF and PES membranes might be worn off.

It should be noted that the pure water fluxes of PVDF and PTFE decreased more significantly, while the pore size change of the PTFE membrane was not very considerable. Since the pore size of the PTFE was much smaller, membrane fouling, in this case, may primarily comprise pore blocking rather than pore constriction. In this case, the fouled pores were completely blocked and the unfouled pores were still open. Pore size analysis could only measure the open pores; thus, the pore size of the used PTFE did not change significantly. However, pore blocking significantly decreased the number of available pores; thus, pure water flux decreased correspondingly. For the PVDF membrane, the pore’s size was much larger; thus, pore blocking and pore constriction may occur concurrently. The decline in pore size and the decrease in flux were both observed. The fouling of the PES membrane was much lower compared to the PVDF and PTFE membranes, possibly due to the more hydrophilic nature of the PES membrane.

The change of membrane properties in the pilot experiment results from both membrane aging and membrane fouling, and identifying the sole effect of membrane aging on membrane properties is difficult, as shown in Figure 1. Thus, batch tests were conducted to elucidate the susceptibility of the different membranes towards chemical cleaning.

### 3.2. Membrane Properties in the Acid Aging Test

**Figure 2:**
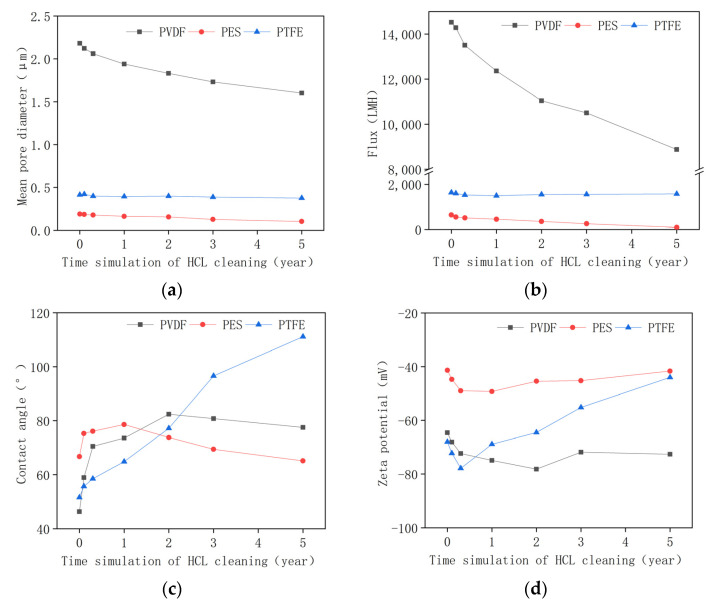
(**a**) Pore size, (**b**) pure water flux, (**c**) contact angle, and (**d**) zeta potential of the pristine membrane and aged membrane in the acid aging test.

Acid soaking and alkaline oxide soaking were conducted separately. As shown in Figure 2a,b, acid chemical cleaning induced a significant decline in pore size and a decrease in pure water flux. This may be attributed to the loss of hydrophilicity [20]. The pore’s size was determined by using capillary flow porometry, which measured the gas pressure required to force a wetting liquid out of through pores. The pore’s size can be calculated using the Washburn equation [33]:

(1) $$D=\frac{4\gamma \cdot cos\theta }{P}$$

where *D* is the pore size, *P* is the pressure measured, *γ* is the surface tension of the wetting liquid, and *θ* is the contact angle of the wall of the pore.

According to the Washburn equation, as the contact angle increases (becoming more hydrophobic), the pore size calculated will decrease. Figure 2c shows that the contact angle of the membrane surface increased in the acid aging procedure. It should be noted that the contact angle shown in Figure 2c corresponded to the membrane’s surface, which was different from the contact angle related to the wall of the pore in Equation (1). Due to the difficulty in determining contact angle of the inner wall, it may be reasonable to assume that the pore wall contact angle may experience the same trend as the membrane’s surface contact angle in aging. In addition, the PTFE membrane is similar to a depth filter, while PVDF and PES membranes are surface filters [34]. The depth filter PTFE membrane possessed higher surface roughness and, thus, may be more susceptible to chemical aging. The significant increases in surface contact angle and surface zeta potential observed for the PTFE membrane indicated that acid chemical cleaning may aggravate its fouling tendency.

The zeta potential of all membranes decreased initially and then increased gradually. The initial decline in the zeta potential may be attributed to the loss of functional groups in aging. The obvious increase in the zeta potential for PTFE may be due to the re-formation of macromolecular cross-linking.

**Figure 3:**
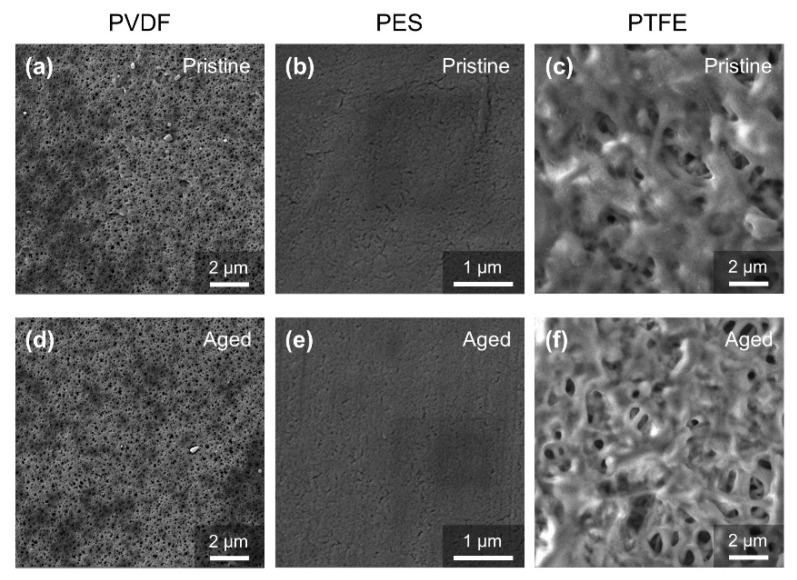
SEM photograph of the pristine membrane and aged (five-years equivalent) membrane in the acid aging test. (**a**) Pristine PVDF membrane, (**b**) prestine PES membrane, (**c**) pristine PTFE membrane, (**d**) aged PVDF membrane, (**e**) aged PES membrane, (**f**) aged PTFE membrane.

Figure 3 shows how surface morphology changed before and after acid chemical soaking. No obvious change in the surface morphology could be found for the three membranes. It was determined that the pore size of the PVDF membrane was much larger than that of the PES membrane, which was consistent with the results of the porometry measurement (Figure 2a). The PTFE membrane demonstrated a multi-layer thread-like structure. Although the surface pore size of the PTFE membrane was the largest, the multi-layer depth filtration structure allowed it to intercept foulants that were much smaller than the surface pore size. The pore size of the PTFE, determined by a capillary flow porometer, also indicated that its apparent pore size was smaller than the PVDF membrane and is comparable to the PES membrane. In addition, the pore size of the pristine membrane and aged membrane did not change significantly for all three membranes, according to the SEM results. This implies that the pore size decline for the PVDF and PES, obtained by a capillary flow porometer as shown in Figure 2a, may be attributed to the loss of hydrophilicity.

**Figure 4:**
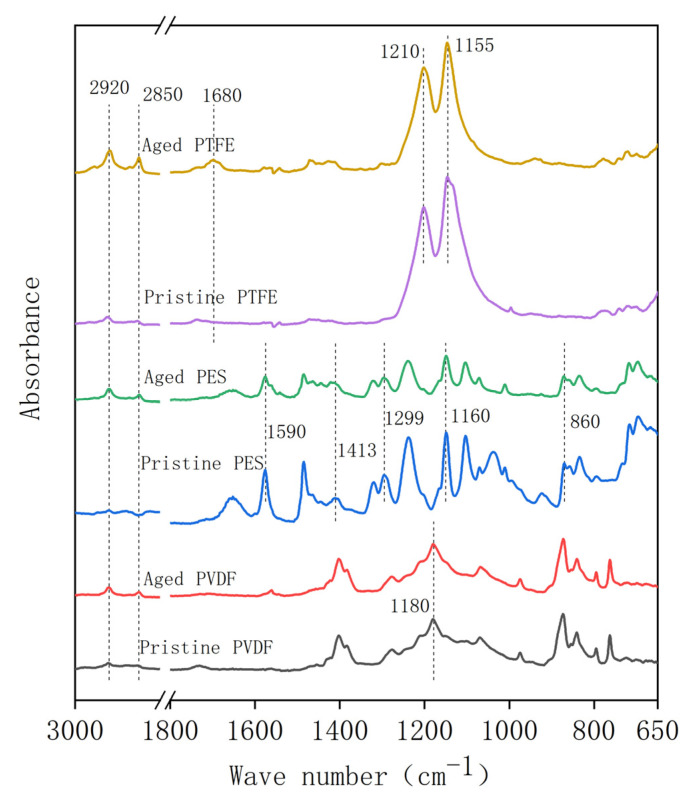
The FTIR of pristine and aged (five-years equivalent) membranes in the acid aging test.

The ATR-FTIR spectra of the pristine and aged membranes are shown in Figure 4. For the PVDF membrane, the characteristic peaks (at 1400, 1280, 1180, and 1072 cm^−1^) did not change significantly in acid aging, while two new peaks at 2850 and 2920 cm^−1^, representing C-H2 and C-H3 bonds, appeared in the aged specimen. This indicated that a fluoro-substitution reaction may occur [35]. These two peaks also appeared in the aged PES and PTFE membrane specimen, which indicated a similar substitution of functional groups on the carbon chain. For the PES membrane, the intensities of the aromatic ring (1590 cm^−1^), C-S bond on the sulfonic group (1413 cm^−1^), C-O group (1299 cm^−1^), sulfuryl group (Ar-SO_2_-Ar, 1160 cm^−1^), and C-H bond on the benzene ring (860 cm^−1^) decreased in the aging procedure. This may be attributed to the chain scission of PES molecules [17]. For the PTFE membrane, an extra peak at 1680 cm^−1^ appeared in the aged membrane, and this peak represented alkenyl C=C stretch, which may result from the dehydrofluorination reaction [35].

### 3.3. Membrane Properties in the Alkaline Oxide Aging Test

**Figure 5:**
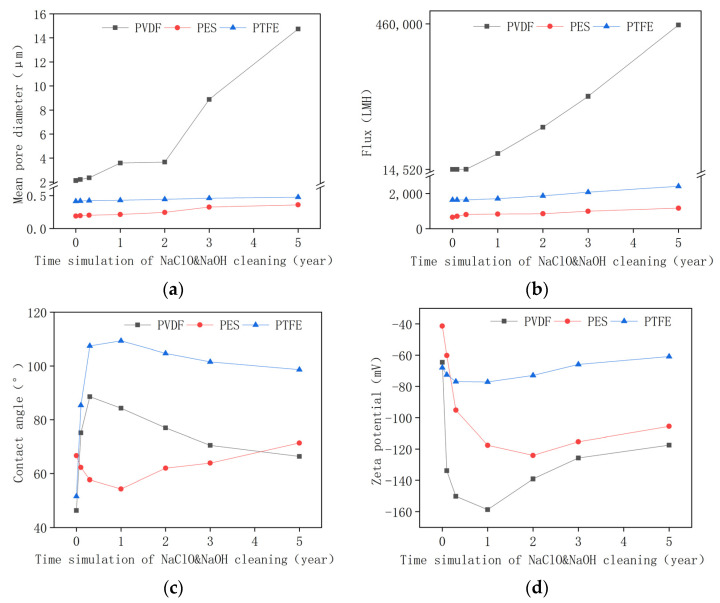
(**a**) Pore size, (**b**) pure water flux, (**c**) contact angle, and (**d**) zeta potential of the pristine membrane and aged membrane in the alkaline oxide aging test.

Sodium hydroxide and sodium hypochlorite were used together to conduct the alkaline oxide aging test for their strong cleaning performance. This combination has been widely used to clean severe membrane fouling, e.g., in textile wastewater treatment [1]. As shown in Figure 5a, with aging, the membrane’s pore size increased to different extents for different membranes, e.g., 2.13 μm to 14.73 μm for PVDF, 0.188 μm to 0.360 μm for PES, and 0.414 μm to 0.478 μm for PTFE. This may be attributed to the oxidation of the membrane additives (such as polyvinyl pyrrolidone, PVP) or to the deterioration of the membrane’s material [17]. The pure water flux of all membranes increased after aging tests (Figure 5b), e.g., from 14,520 LMH to 456,600 LMH for the PVDF membrane, from 642 LMH to 1164 LMH for the PES membrane, and from 1640 LMH to 2420 LMH for the PTFE membrane. Pure water flux changed consistently with pore size variations. The PVDF membrane was the most susceptible to alkaline oxide aging; the pore’s size increased by 5.92 folds and flux increased by 30.45 folds [36]. The PTFE membrane was rather stable against caustic oxide aging, with pore size increases by 15.46% and flux increases by 47.56%. This trend is different from acid aging, which induced decreases in pore size and flux. In acid aging, the structure of the membrane and pore remains intact, and interfacial interaction may be the major factor that determined the pore’s size and flux, while in caustic oxide aging, serious erosion and pore enlargement may occur [36,37].

Figure 5c shows the contact angle of the pristine and aged membranes. The rapid increase in the contact angle in the beginning may be attributed to the wash out of the hydrophilic coating or grafting. The subsequent decrease in contact angle for the PVDF and PTFE membrane could be attributed to the oxidation or erosion of the membrane material (e.g., dehydrofluorination process), which resulted in a more hydrophilic surface. For the PES membrane, the contact angle experienced a decline followed by a gradual rise. The initial decrease could be ascribed to the formation of phenol groups and hydrolysis of the sulfonyl group, and the subsequent increase may be attributed to the degradation of hydrophilic additives (such as PVP) [38].

The zeta potential of all membranes showed a down-and-up trend. The initial decrease in zeta potential may be attributed to the dehydrofluorination reaction in PVDF and PTFE aging and the formation of phenol group and sulfonic acid functions in PES aging. The subsequent increase in the zeta potential may result from the reformation of macromolecular cross-linking.

**Figure 6:**
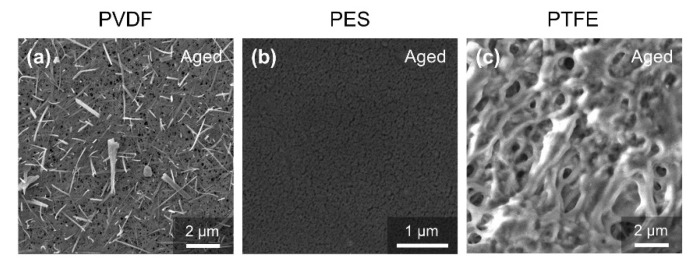
SEM photograph of the pristine membrane and aged (five-years equivalent) membrane in the alkaline oxide aging test. (**a**) Aged PVDF membrane, (**b**) aged PES membrane, (**c**) aged PTFE membrane.

**Figure 7:**
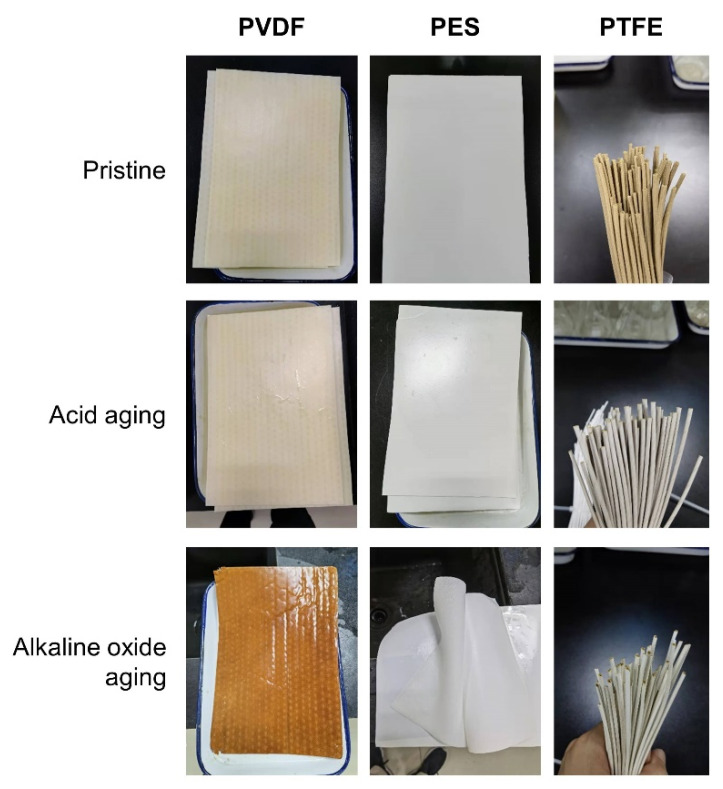
Photographs of the pristine and aged (five-years equivalent) membrane in the batch aging test.

The surface morphology of the aged membrane is shown in Figure 6. The PVDF membrane was seriously eroded, with an appearing fiber-like supporting layer. This could also explain the drastic increases in pore size and flux in the PVDF membrane. No obvious change could be found for the PES and PTFE membranes. Photos of pristine, acid-aged, and alkaline oxide-aged membranes are shown in Figure 7. The alkaline oxide-aged PVDF membrane turned red and brittle, which indicated severe aging and dysfunction. The susceptibility of PVDF to alkaline oxide chemicals has also been reported [17,20,35,36].

**Figure 8:**
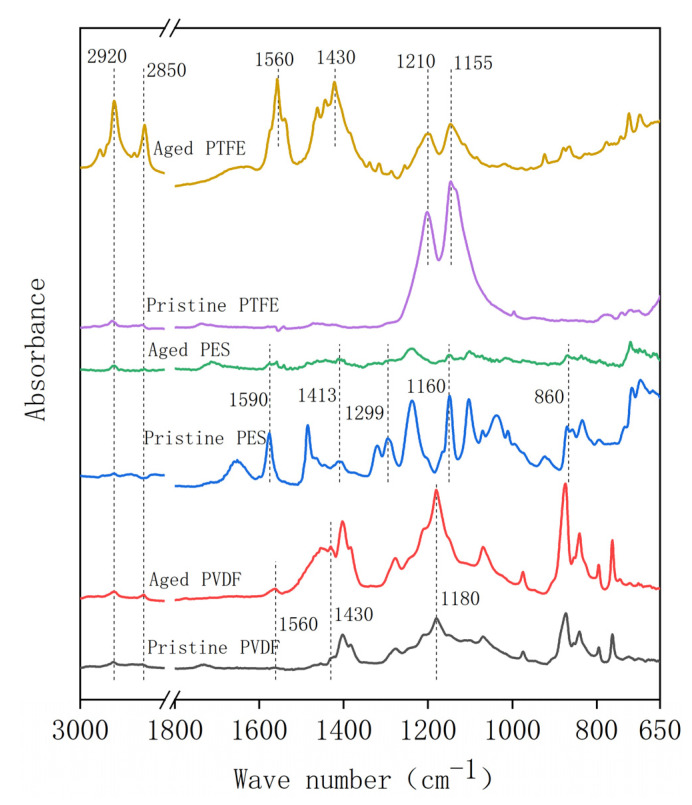
The FTIR of pristine and aged (five-years equivalent) membranes in the alkaline oxide aging test.

According to the FTIR analysis (Figure 8), the C-H_2_ (2850 cm^−1^) and C-H_3_ (2920 cm^−1^) stretching peaks appeared in the alkaline oxide aging for all three membranes, which could be attributed to the substitution reaction and the formation of alkane. For the PVDF membrane, the peak appeared at 1430 cm^−1^, indicating the generation of C-OH bending in carboxylic acid, and the peak that appeared at 1560 cm^−1^ peak (C=C bond) evidenced dehydrofluorination reactions. These reactions enhanced the hydrophilicity and electronegativity of the membrane. For the PES membrane, the scission of (Ph-SO2-Ph-O)_n_ chain could be verified by the downward trend of all characteristic peaks of PES [38]. For the PTFE membrane, peaks at 1210 cm^−1^ and 1155 cm^−1^, representing the CF_2_ bond, decreased, while peaks at 1430 cm^−1^ and 1580 cm^−1^, representing the C=C bond, increased, indicating a dehydrofluorination reaction.

Unlike acid cleaning, which only altered the interfacial property of membranes, alkaline oxide cleaning induced much more severe membrane aging or erosion. Sodium hydroxide and sodium hypochlorite are the chemicals most commonly used in the removal of organic fouling. Particularly for MBR for textile wastewater treatments, frequent alkaline oxide cleaning may be indispensable. Therefore, special anti-aging modifications for membranes susceptible to chemical cleaning (such as PVDF) would be necessary for the MBR for textile wastewater treatment; otherwise, membranes that are rather stable under intensive chemical cleaning (like PES or PTFE) should be chosen.

## 4. Conclusions

This work investigated the aging of three different membranes—PVDF, PES, and PTFE—used in MBR for textile wastewater treatment. Pilot-scale MBRs were carried out, and the used membrane was studied to investigate fouling and aging. Batch aging tests were conducted to elucidate the aging process of three membranes. The following conclusion could be drawn:

1. The PVDF membrane was most susceptible to chemical cleaning. In the acid aging test, the surface hydrophobicity of PVDF increased, and the pore size and the pure water flux decreased due to the elevated hydrophobic effect. Alkaline oxide aging destructed the PVDF membrane’s structure, enlarged pore size, and increased pure water flux. The fluoro-substitution reaction and the dehydrofluorination reaction may occur in the aging.
2. The PES and PTFE membranes were rather stable. Chemical cleaning barely changed the surface structure of the membrane specimens, although the interfacial properties (hydrophobicity and surface zeta potential) were altered. The chain scission of PES molecules and the dehydrofluorination of the PTFE were observed in aging.
3. Membrane aging in the MBR for textile wastewater treatment should be carefully considered due to the possible intensive chemical cleaning process.

## Figures and Tables

**Table 1:** Water quality of the influent of pilot-scale MBRs.

	COD_cr_ (mg/L)	Ammonia Nitrogen (mg/L)	Total Phosphorus (mg/L)	pH
Low loading From the exit end of the aeration tank	145 ± 23	0.26 ± 0.12	0.41 ± 0.31	7.98 ± 0.53
Medium loading From the middle of the aeration tank	187 ± 35	0.89 ± 0.26	1.94 ± 1.13	8.12 ± 0.29
High loading From the inlet of the aeration tank	560 ± 91	17.35 ± 3.84	6.10 ± 2.93	8.29 ± 0.65

**Table 2:** Parameters of the membrane modules and the corresponding MBRs.

	Membrane Material	Membrane Module	Membrane Area (m^2^)	Flux (LMH)	Operation Mode (min)	MLSS in MBR Tank (g/L)
MBR 1	PVDF	Flat sheet	420	15	8 min on, 2 min off	12
MBR 2	PES	Flat sheet	470	15	8 min on, 2 min off	12
MBR 3	PTFE	Hollow fiber	360	15	8 min on, 2 min off	12

**Table 3:** Calculation of CT values of cleaning frequency, cleaning duration, and chemical concentration.

Chemical	Chemical Cleaning Procedures in Real MBR Operation		CT Value (gh/L)	Batch Tests						
	Offline Cleaning (1 per Month for 12 h)	Online Cleaning (1 per Week for 15 min)	1 Month	Chemical Concentration	Soaking Time in a Continuous Batch-Test Matched with the CT Value in Real MBRs Clean					
					1-Month Equivalent	3-Months Equivalent	1-Year Equivalent	2-Years Equivalent	3-Years Equivalent	5-Years Equivalent
Sodium Hypochlorite (mg/L)	3000	500	36.5	3000	12.16 h	36.48 h	6.08 days	12.16 days	18.24 days	30.40 days
Sodium hydroxide (mg/L)	40,000		480	39,452	12.16 h	36.48 h	6.08 days	12.16 days	18.24 days	30.40 days
Hydrochloric acid (mg/L)	20,000	500	240.5	19,767	12.16 h	36.48 h	6.08 days	12.16 days	18.24 days	30.40 days

## Data Availability

Data are available on demand to the authors.
